# ZnO nanoparticles efficiently enhance drought tolerance in *Dracocephalum kotschyi* through altering physiological, biochemical and elemental contents

**DOI:** 10.3389/fpls.2023.1063618

**Published:** 2023-03-10

**Authors:** Zahra Karimian, Leila Samiei

**Affiliations:** Department of Ornamental Plants, Research Center for Plant Sciences, Ferdowsi University of Mashhad, Mashhad, Iran

**Keywords:** antioxidant enzyme activity, drought stress, nanoparticles, zinc fertilizers, elemental composition

## Abstract

Using nanofertilizers in certain concentrations can be a novel method to alleviate drought stress effects in plants as a global climate problem. We aimed to determine the impacts of zinc nanoparticles (ZnO-N) and zinc sulfate (ZnSO_4_) fertilizers on the improvement of drought tolerance in *Dracocephalum kotschyi* as a medicinal-ornamental plant. Plants were treated with three doses of ZnO-N and ZnSO_4_ (0, 10, and 20 mg/l) under two levels of drought stress [50% and 100% field capacity (FC)]. Relative water content (RWC), electrolyte conductivity (EC), chlorophyll, sugar, proline, protein, superoxide dismutase (SOD), polyphenol oxidase (PPO) and, guaiacol peroxidase (GPO) were measured. Moreover, the concentration of some elements interacting with Zn was reported using the SEM-EDX method. Results indicated that foliar fertilization of *D. kotschyi* under drought stress with ZnO-N decreased EC, while ZnSO_4_ application was less effective. Moreover, sugar and proline content as well as activity of SOD and GPO (and to some extent PPO) in treated plants by 50% FC, increased under the influence of ZnO-N. ZnSO_4_ application could increase chlorophyll and protein content and PPO activity in this plant under drought stress. Based on the results, ZnO-N and then ZnSO_4_ improved the drought tolerance of *D. kotschyi* through their positive effects on physiological and biochemical attributes changing the concentration of Zn, P, Cu, and Fe. Accordingly, due to the increased sugar and proline content and also antioxidant enzyme activity (SOD, GPO, and to some extent PPO) on enhancing drought tolerance in this plant, ZnO-N fertilization is advisable.

## 1 Introduction

According to climate change and changes in global precipitation patterns, predictions and models have shown an increase in the size of deserts and xeric scrublands as the largest terrestrial biome (Guo et al., 2021). Every year, water scarcity and drought cause severe damage to agricultural products, green spaces, forests, and rangelands. In the past few years, these issues have resulted in a global decline in agricultural production (Bodner et al., 2015). In addition, water scarcity and drought contribute to the inhibition of crop growth and negatively impact plant quantitative and qualitative yield, as well as physiology and morphology (Bayat and Moghadam, 2019).

Applying fertilizers and nutritional compounds during the growth state is a crucial strategy to decrease the impact of drought on crops. Several studies have shown that micronutrients mitigate crops drought stress (Adrees et al., 2020; Bashir et al., 2020; Dimkpa et al., 2020). Zinc is a microelement, necessary for many plant activities, which plays a crucial role in protein, enzyme, and chlorophyll synthesis and the improvement of agricultural crop yield (Singh et al., 2018; Dimkpa et al., 2019). Furthermore, studies have confirmed the effect of Zn, in the form of zinc sulfate, zinc chelate, and Zn nanoparticle, on enhanced resistance to environmental stress by improving morphological, physiological, and biochemical factors in different plants (Mahdieh et al., 2018; Farooq et al., 2020; Faizan et al., 2021; Tariverdizadeh et al., 2021).

Nanoparticles (particles that are ≤100 nm in at least one dimension) play an essential role in altering different physiological processes in plants, nutrient absorption, plant growth, and increased plant resistance to abiotic stress (Jordan et al., 2018; Dimkpa et al., 2020). Moreover, nanoparticles are used in the form of micronutrients and fertilizers to improve qualitative and quantitative crop yield. Because of the limited and controlled release of nutrients, nanoparticles can contribute to plant growth and resistance to different types of stress (Adrees et al., 2020; Bashir et al., 2020).

Zn nanoparticle is a new plant fertilizer produced with the technology of nanoparticle synthesis, which has many benefits, such as increased tolerance to abiotic stress in some plants (Cicek and Nadaroglu, 2015; Rossi et al., 2018; Singh et al., 2018; Zhang et al., 2018; Farooq et al., 2020; Taghizadeh et al., 2020; Faizan et al., 2021; Tariverdizadeh et al., 2021). Studies have reported the positive effects of Zn nanoparticles on improved tomato (Faizan et al., 2021) and wheat (Bashir et al., 2021) yield under salinity and drought stress, respectively. In addition, Dimkpa et al. (2019) and Motyka et al. (2019) have approved increased yield of sorghum plants under drought stress and enhanced the situation of bryophytes under oxidative stress with the help of Zn nanoparticles. Moreover, different forms of zinc were applied to the coffee plant, compared to zinc sulfate, Zn nanoparticles could positively affect coffee plant growth and yield (Rossi et al., 2018).

Element interactions in plants can affect plant functions, such as growth, yield, and stress resistance. Studies have suggested that the Zn element has antagonistic and synergistic interactions with Cu, Fe, and P elements in absorption, transfer, and chemical reactions in the plant. It may vary depending on factors such as plant type, soil type, nutrition, and weather conditions (Fageria et al., 2012; Izsáki, 2014; Rietra et al., 2017). The impact of element interactions to nanoparticle form on abiotic stress resistance (e.g., drought) has been reported in some studies (Liu et al., 2020; Ahmed et al., 2022).

*Dracocephalum kotschyi* belongs to Lamiaceae and is one of the 60 species of the genus *Dracocephalum*. This species is endemic to Iran and is currently considered an endangered plant. *D. kotschyi* is known to have several medicinal properties (Muaffarīyān, 1996). This species is traditionally a medicine plant in Iran with several therapeutic effects such as analgesic, antispasmodic, and anticancer (Sharafi et al., 2014). *D. kotschyi* also was used in the treatment of headaches, congestion, stomach and liver disorders (Dorosti and Jamshidi, 2016). In addition to various pharmacological effects, it is used in industry, medicine, and food as a source of some valuable secondary metabolites. The most important secondary metabolites of this specimen include monoterpene glycosides, trypanocidal terpenoids (Saeidnia et al., 2004), flavonoids, rosmarinic acid (Fattahi et al., 2013), and some essential oils (Saeidnia et al., 2007). This herbaceous, beautiful, and aromatic wild-growing plant with a height of about 10-20 cm is grown in mountainous and highland areas of the central and northern regions of the country (Rechinger, 1986). In addition, *D. kotschyi* is used as an ornamental plant due to its aesthetic characteristics, such as abundant white and fragrant flowers, plant form, relatively fast growth, long flowering stage, and possible abiotic stress tolerance. Therefore, it can be used as a valuable ornamental plant in sustainable green space design in arid and semi-arid areas.

Climate change, especially consecutive years of drought, has been one of the critical extinctions treat of this valuable medical-ornamental plant. Notably, that most studies performed on this species have focused more on its phytochemical attributes, and few studies are available on strategies to enhance the abiotic stress tolerance of this species which could contribute to the cultivation development and survival of this plant.

The present study aimed to determine the effect of different concentrations of zinc in two forms of zinc oxide (ZnO-N) nanoparticles and zinc sulfate (ZnSO_4_) on the drought tolerance of *D. kotschyi* plant. It is hypothesized that ZnO-N compared to ZnSO_4_ might be more effective in alleviating the drought tolerance of *D. kotschyi* through its positive function on the physiological and biochemical processes of the plant.

## 2 Materials and methods

### 2.1 Plant growth conditions and treatments

Mature seeds of *D. kotschyi* were collected from its natural habitat in Semirom city (Isfahan province, Iran) with latitude and longitude of 31°49’, 51° 59’ “, respectively, and altitude of 2400 m above sea level. Seeds were grown in growth trays containing 80% cocopeat and 20% perlite. After germination, seedlings in the six-leaf stage were transferred to plastic pots containing sandy loam soil with a low organic matter (1.5%) and a pH of 7.2. Following ten days of seedling transplanting, potted plants were regularly (twice a week) fed with an NPK fertilizer (20-20-20, 2g/l). After three weeks of growth in optimal conditions in the greenhouse, seedlings were divided in two groups: the first group (control) under well-watering (100% field capacity (FC)) and the second one (drought-stressed group) under limited watering (50% field capacity). According to the gravimetric method, soil moisture content was measured (Datta et al., 2009). It was monitored daily during the growth period using a soil moisture meter (EXTECH MO750, USA, probe length: 20 cm probe and max resolution: 0.1%). The pots are watered (when the humidity dropped below a certain level) to keep the moisture content at the desired level (100% and 50% FC).

Foliar fertilization with ZnSO_4_ and ZnO-N at three concentrations of 0 (control), 10, and 20 mg/l was performed two times; first simultaneously with the start of drought stress and second three weeks after the start of drought stress (Rossi et al., 2018; Azmat et al., 2022). The control plants were watered up to 100% FC and received no fertilizer treatments (ZnSO_4_ and ZnO-N).

**Figure 1:**
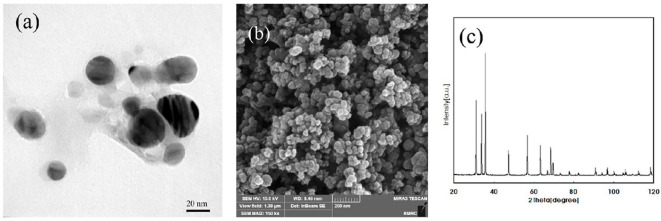
**(A)** Transmission Electron Micrograph (TEM), **(B)** SEM (Scanning electron microscope) and **(C)** X-ray diffraction (XRD) of Zinc oxide nanoparticles, Iranian Nanomaterials Pioneers Company, NANOSANY, 2020.

ZnO-N were procured from Iranian Nanomaterials Pioneers Company, NANOSANY (Mashhad, Iran). Its characteristics, such as particle size, and purity, are presented in **Figure 1** and **Supplementary File 1**. ZnO-N were added to deionized water and dispersed using ultra-sonication for 30 minutes. The treated plants were collected 45 days after the start of drought stress treatment, and leaf samples were immediately placed in liquid nitrogen and stored in a -80 freezer for later analysis.

### 2.2 Electrolyte conductivity and relative water content measurement

In order to measure EC, one gram of each leaf was added to 20 ml of distilled water and kept at room temperature (24°C) for 24 h. In addition, its electrical conductivity was measured by an EC meter as the initial EC (EC_1_). After that, the samples were placed in an autoclave at 120°C for 15 min, and their secondary EC (EC_2_) was determined. The following equation was exploited to calculate EC.


$$\text{EC }=\;{\text{(EC}}_{1}/{\text{EC}}_{2})\times 100$$

Fresh weight (FW) of fully mature leaves was measured immediately after collection based on a method by (Ritchie et al., 1990). Afterward, they were placed in distilled water at 4°C for 24 h to determine the turgor weight (TW). In the next stage, samples were dried in an oven at 70°C for 48 hours, and the dry weight (DW) of each sample was recorded. The percentage of RWC was estimated using the equation below:


RWC = [(FW−DW)/(TW−DW)×100]

### 2.3 Photosynthetic pigments and soluble sugar determination

Leaf samples homogenized with 80% acetone were centrifuged for 5 min at 3000×g to calculate photosynthetic pigments (chlorophyll and carotenoid). Following that, optical absorbance of the supernatant solution was read at wavelengths of 470, 645, and 663 nm using a spectrophotometer (UV-VIS, Optima SP-3000 Plus, Bratislava, Slovakia). In the next stage, total chlorophyll and carotenoids were calculated in mg/g FW (Lichtenthaler, 1987).

Soluble sugar was determined using a phenol-sulfuric acid method based on acid hydrolysis of soluble sugars and furfural mixture creation (Kochert, 1978). After that, 100 µL of ethanol extract from the leaf sample, 300 µL of distilled water, and 1 mL 5% phenol were mixed while vortexed. In the next stage, 1 mL of concentrated sulfuric acid was added to the mixture, and adsorption was read at 485 nm against blanks after 30 min. In addition, the sugar content of the samples was assessed by using standard curves in mg/g FW.

### 2.4 Proline content determination and assay of total protein

Based on the technique by Bates et al. (1973), 200 µL of ninhydrin and glacial acetic acid reagents was added to 200 µL of extract from homogenized leaf sample and sulfuric acid. Afterward, the samples were placed in a hot water bath at a temperature of 100°C for 30 min. Then, 600 µL of toluene was added to the samples following immediate rapid cooling and vortexed for 30 sec. After 20 minutes, the optical absorbance of the upper solution was read at 520 nm, and the concentration of proline in the solution was calculated in mg/g FW using the proline standard curve.

In order to assay total protein, 1 mL of extraction buffer (PBS) and EDTA with a concentration of 0.1 mM was added to powdered leaves using liquid nitrogen and homogenized. After that, the samples were centrifuged at 12000g for 10 minutes at 4°C. The supernatant was stored in a freezer at -80°C for protein and enzyme activity assay. In the next stage, 2.5 mL of Bradford solution was added to 50 µL of supernatant to determine the protein content of the extract. After 5 min, adsorption was read at a wavelength of 595 nm. Certain concentrations of bovine serum albumin (BSA) standard protein were prepared in order to draw a standard protein diagram (Bradford, 1976).

### 2.5 Assay of antioxidant enzyme activity

In this stage, 50 mM of PBS (pH=7), 0.02 M pyrogallol, and 50 µL enzymatic extract were prepared as the reaction mixture to assess the activity of polyphenol oxidase (PPO). Adsorption of all samples was measured based on the purpurogallin content at a wavelength of 420 nm in one min at a 5 second interval., Ultimately, the values obtained were calculated in Umg^-1^ protein (Raymond et al., 1993).

Superoxide dismutase (SOD) enzyme activity in the reaction mixture was assessed based on the measurement of inhibition of photochemical reduction of nitro blue tetrazolium. Reaction mixture encompasses BPS (50 mM, pH=7.8), methionine (13 mM), nitro blue tetrazolium (75 µM), riboflavin (2 µM), sodium carbonate (50 mM), triton X-100 (0.025%) and 50 µL of enzymatic extract. Sample adsorption was read at a wavelength of 560 nm against the blanks following exposure to light for 15 min. In addition, Blue Formazan production was expressed in Umg^-1^ protein by measuring the increase in absorption (Giannopolitis and Ries, 1977).

Guaiacol peroxidase (GPO) enzyme activity was assessed using (Hemeda and Klein, 1990) method. The reaction mixture included BPS (20 mM, pH=6), Guaiacol (5 mM), H_2_O_2_ (1 mM), and 50 µL of enzymatic extract. In addition, enzyme activity resulting from guaiacol oxidation with increased adsorption at 570 nm was determined. Alternation in adsorption was measured every five seconds for a minute and was expressed in Umg^-1^ protein.

### 2.6 SEM-elemental mapping and EDX analysis

Following the method by Pathan et al. (2010), intact and mature leaf samples separated from plants were powdered after drying at room temperature without any pretreatment and were used for SEM imaging. In addition, microanalysis of Zn, as well as Cu, Fe, and P elements as the most critical interactive elements with Zn, and elemental mapping was carried out using SEM-EDX (Energy-Dispersive X-ray). It is worth mentioning that all microscopic studies were done at Bu-Ali Research Institute, Mashhad University of Medical Sciences.

### 2.7 Statistical analysis

In the present study, a factorial experiment was conducted based on the completely randomized design with four replications (each pot represented one replication with two plants). Treatments were drought stress, including 50% FC and 100% FC and two zinc sources, ZnSO_4_ and ZnO-N, at three concentrations of 0, 10, and 20 mg/l. The distribution normality of data was tested using the Anderson-Darling test prior to analysis. Analysis of variance (ANOVA) and means the comparison of measured attributes was performed using Minitab 16 software and Tukey’s test at 5% probability level, respectively.

## 3 Results

### 3.1 EC and RWC measurement

**Figure 2:**
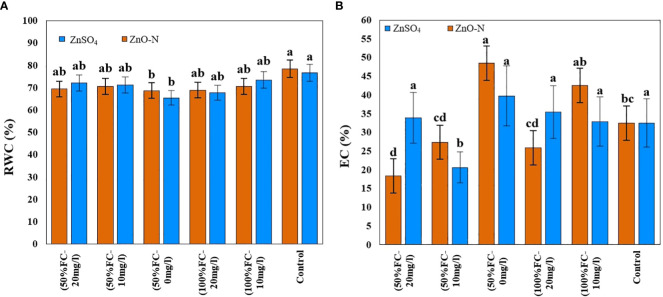
Impact of ZnSO_4_ and ZnO-N on RWC **(A)** and EC **(B)** in *D Kotschyi* under Drought Stress. Means followed by the same letters did not differ significantly at p ≤ 0.05, n = 4.

It is evident from **Table 1** that ZnSO_4_ and ZnO-N and their interactions with drought stress had significant effects on EC in *D. kotschyi* (P ≤ 0.05) (**Table 1**). In this respect, the EC of the samples (22.12%) decreased by 83%, compared to control (40.47%), by increasing the ZnO-N concentration. The lowest EC was observed in samples under drought stress and without drought stress receiving 20 mg of ZnO-N (18.4% and 25.84%). Foliar fertilization with ZnSO_4_ at a concentration of 10 mg/l had a mitigating effect on the EC, such that the lowest EC was obtained in plants under drought stress and fertilized by 10 mg/l of ZnSO_4_ (20.62%) (**Figure 2**, right).

**Table 1:** Analysis variance of physiological and biochemical traits in treated *D. kotschyi* by ZnSO_4_ and ZnO-N under drought stress.

P-Value										
Source	DF	RWC	EC	Chlorophyll	Sugar	Proline	Protein	SOD	GPO	PPO
(DS)	1	0.096	0.306	0.226	0.491	0.000	0.344	0.004	0.000	0.032
(ZnSO_4_)	2	0.553	0.002	0.975	0.026	0.000	0.237	0.000	0.086	0.046
(ZnO-N)	2	0.137	0.000	0.349	0.412	0.286	0.000	0.000	0.000	0.111
(DS)×(ZnSO_4_)	2	0.005	0.002	0.015	0.572	0.553	0.007	0.153	0.260	0.033
(DS)×(ZnO-N)	2	0.038	0.000	0.133	0.000	0.037	0.342	0.000	0.002	0.053
Error	44	–	–	–	–	–	–	–	–	–
Lack of fit	8	0.127	0.000	0.005	0.006	0.000	0.051	0.001	0.026	0.002
Total	52	–	–	–	–	–	–	–	–	–

DS, Drought stress; DF, degree of freedom.

Interaction of foliar fertilization of ZnSO_4_ and ZnO-N with drought stress had a significant effect on changes in RWC content in *D. kotschyi* (P ≤ 0.05) (**Table 1**). At both concentrations of 10 and 20 mg/l, ZnSO_4_ increased RWC in plants under drought stress by up 10%, compared to plants under drought stress not treated with ZnSO_4_ (**Figure 2**, left). This increase was about 3% in plants under drought stress fertilized with ZnO-N at two 10 and 20 mg/l concentrations, although these mitigating changes were not statistically significant. In addition, the highest RWC was related to control plants, which received no treatments, under full irrigation (78.46%) (**Figure 2**, left).

### 3.2 Photosynthetic pigment and soluble sugar content

**Figure 3:**
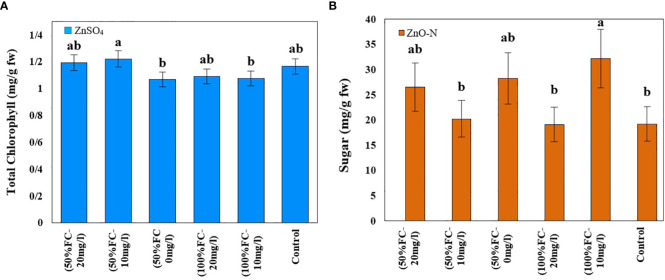
Impact of ZnSO_4_ on total chlorophyll **(A)** and ZnO-N on sugar **(B)** in *D Kotschyi* under Drought Stress. Means followed by the same letters did not differ significantly at p ≤ 0.05, n = 4.

The highest total chlorophyll content was obtained in plants under drought stress and foliar fertilization with ZnSO_4_ at concentrations of 10 (1.22 mg/g FW) mg/l and then 20 (1.20 mg/g FW) mg/l. The lowest amount of chlorophyll (1.07 mg/g FW) was observed in plants under drought stress and without zinc treatment. Moreover, the highest level of carotenoids (2 mg/g FW) was obtained in plants under drought stress. The other treatments had no significant effects on these contents (**Table 1**; **Figure 3**, left).

Drought stress and ZnO-N interaction had a significant effect on soluble sugar in leaf samples of *D. kotschyi* (P ≤ 0.05) (**Table 1**). In addition, the highest sugar content (32.23 mg/g FW) was observed in plants under full irrigation treated with 10 mg/l of ZnO-N. Meanwhile, no significant difference was observed between these samples and the plants under drought stress treated with 20 mg/l of ZnO-N. In this regard, the lowest sugar content (19.16 mg/l FW) was observed in control samples and plants under full irrigation treated with 20 mg/l of ZnO-N (**Figure 3**, right).

### 3.3 Proline and protein content

**Figure 4:**
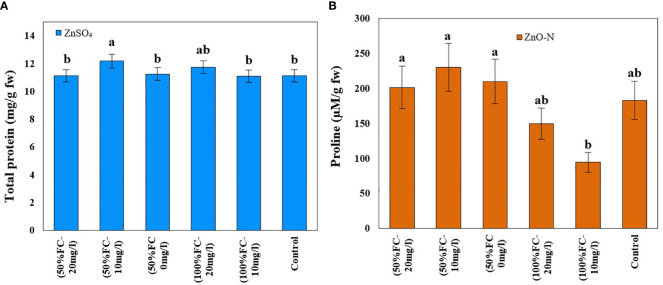
Impact of ZnSO_4_ on total protein **(A)** and ZnO-N on proline **(B)** and in *D Kotschyi* under Drought Stress. Means followed by the same letters did not differ significantly at p ≤ 0.05, n = 4.

In this study, drought stress, ZnSO_4_ and interaction of drought stress with ZnO-N significantly affected proline content in *D. kotschyi* (P ≤ 0.05) (**Table 1**). According to the results, proline content increased in plants under drought stress (213.87 µM/g FW) 50% more than plants under non-stressed conditions (142.32 µM/g FW). Furthermore, ZnSO_4_ fertilization at a concentration of 10 mg/l (233.09 µM/g FW) led to a 136% increase in proline content, compared to plants not treated with this substance (98.67 µM/g FW). According to the results, the highest proline content (230.13 µM/g FW) was observed in plants under drought stress receiving ZnSO_4_ fertilization at a concentration of 10 mg/l, which had a significant difference with plants under full irrigation (no drought stress) (**Figure 4**, left).

ZnO-N and also interaction of ZnSO_4_ with drought stress had a significant effect on the protein content of *D. kotschyi* (P ≤ 0.05). In this regard, the highest protein content (12.02 mg/g FW) was observed in the treated plants with 10 mg/l ZnSO_4_ under drought stress. The lowest one was obtained in plants with 10 mg/l ZnSO_4_ under full irrigation (**Figure 4**, right).

### 3.4 Activity of antioxidant enzymes

**Figure 5:**
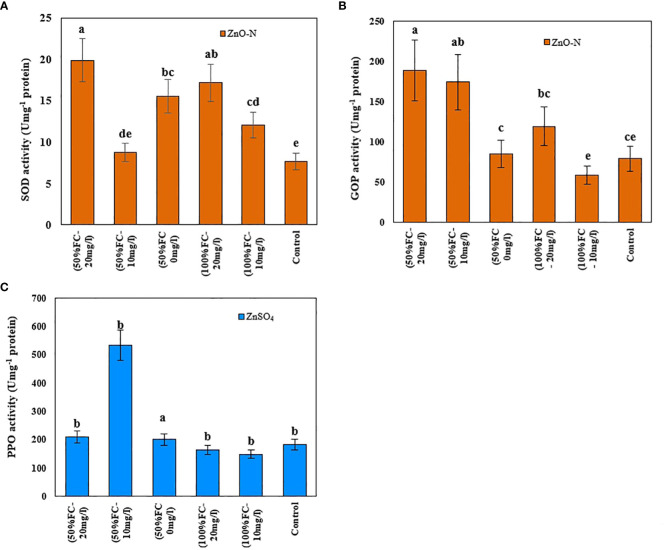
Impact of ZnO-N on SOD **(A)** and GPO **(B)**; ZnSO_4_ on PPO **(C)** in *D Kotschyi* under Drought Stress. Means followed by the same letters did not differ significantly at p ≤ 0.05, n = 4.

In this study, the activity levels of SOD, PPO, and GPO enzymes in *D. kotschyi* under drought stress increased by 20%, 90%, and 75%, respectively, compared to control samples. According to the results, the highest SOD and GPO enzyme activities were observed in plants under drought stress treated with 20 mg/l ZnO-N (**Figure 5**). Meanwhile, the use of 10 mg/l ZnO-N in plants under drought stress led to the highest activity of the PPO enzyme (**Figure 5**, right-above) under Drought Stress

### 3.5 Identification of the elemental composition

SEM-EDX analysis and elemental mapping were conducted to support our findings that Zn-nanoparticles could improve the drought tolerance in *D. kotschyi* by changing the concentrations of Cu, Fe, and P. As seen in **Table 2**, Fe and Cu percentages decreased with a decline in the concentration of Zn in plants, while an increase was observed in P percentage. According to **Table 3**, the concentrations of Zn, Cu, and Fe elements were respectively 5.5%, 5%, and 2.5% higher and the P element was 13% lower in plants treated with ZnO-N and ZnSO_4_, compared to control plants. In addition, the concentration of Cu, Fe, and Zn elements in ZnSO_4_ treatment was 1.5% higher, compared to ZnO-N, whereas the difference in P element concentration was 3.5% lower in this regard (**Tables 2**, **3**).

**Table 2:** Scanning electron microscopy images, Zn maps of leaf surfaces and their EDX results of non-fertilized (control) and foliar fertilized with ZnSO_4_ and ZnO-N in *D. kotschyi* under drought stress.

Source	Zn map	SEM image	EDX Analysis
Control	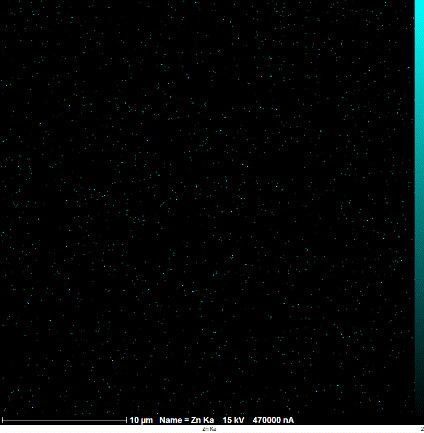	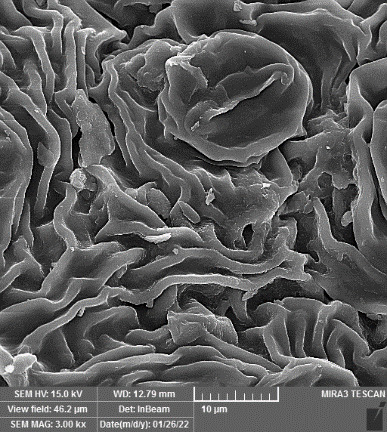	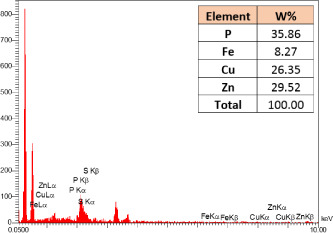
50% FC 10 mg ZnSO_4_	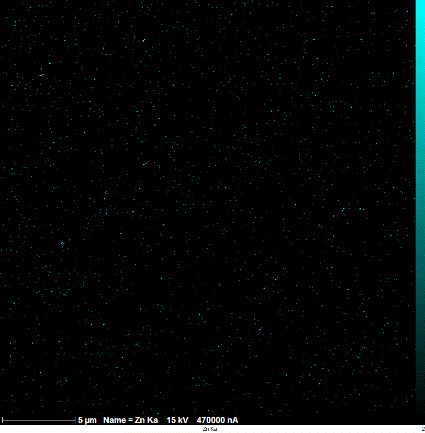	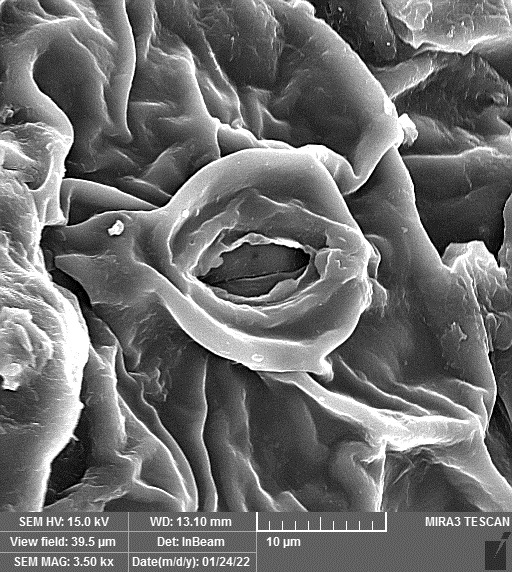	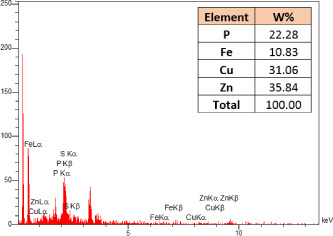
50% FC 20 mg ZnSO_4_	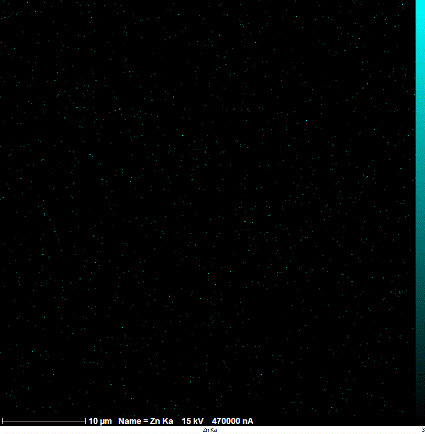	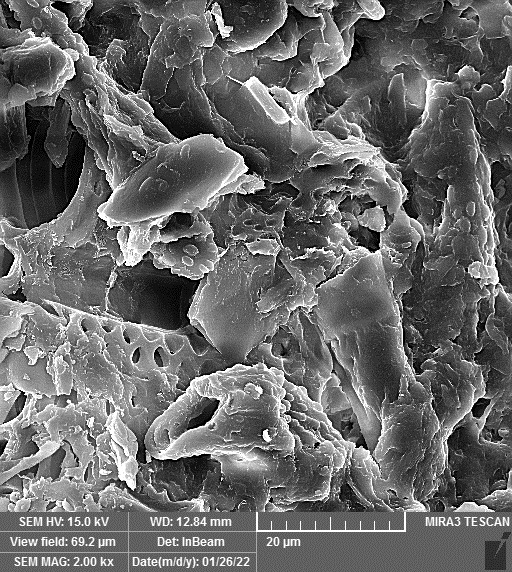	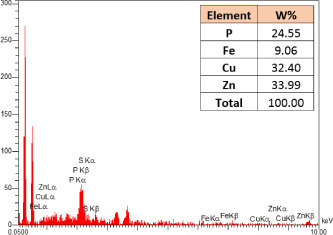
50% FC 10 mg ZnSO_4_ 10 mg ZnO-N	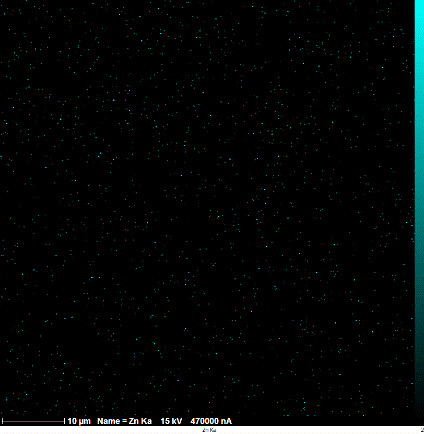	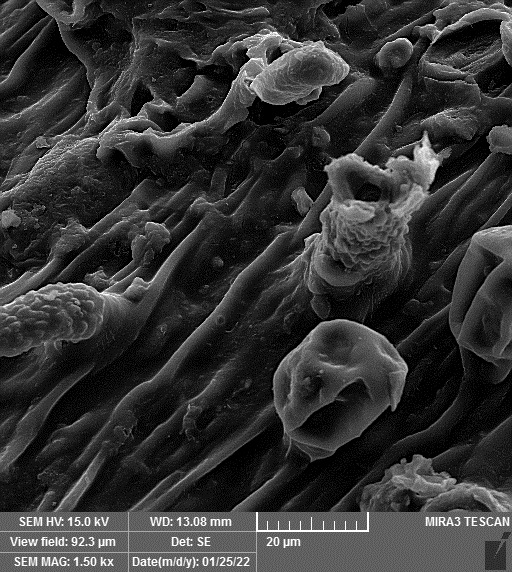	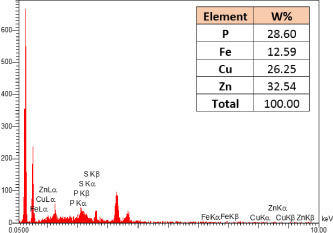
50% FC 20 mg ZnSO_4_ 10 mg ZnO-N	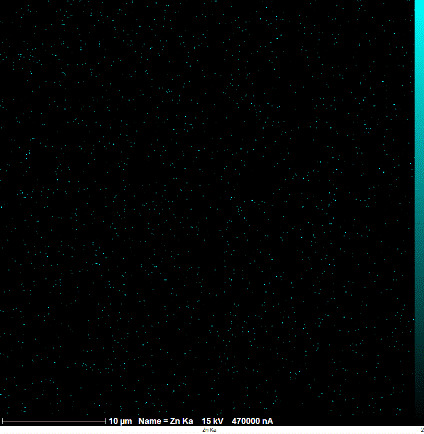	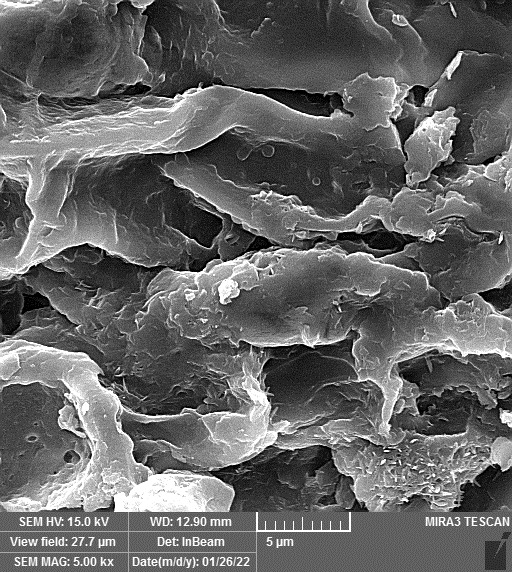	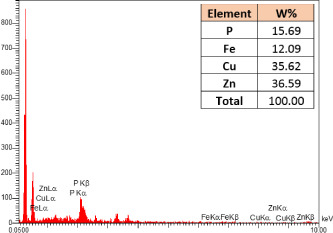
50% FC 10 mg ZnO-N	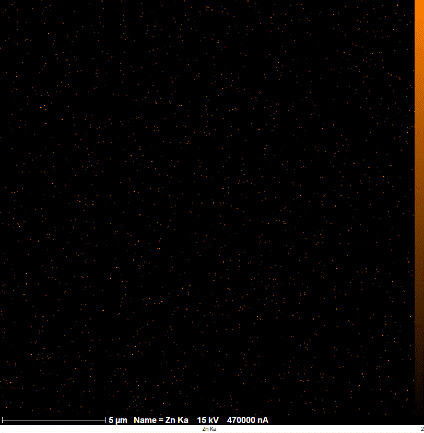	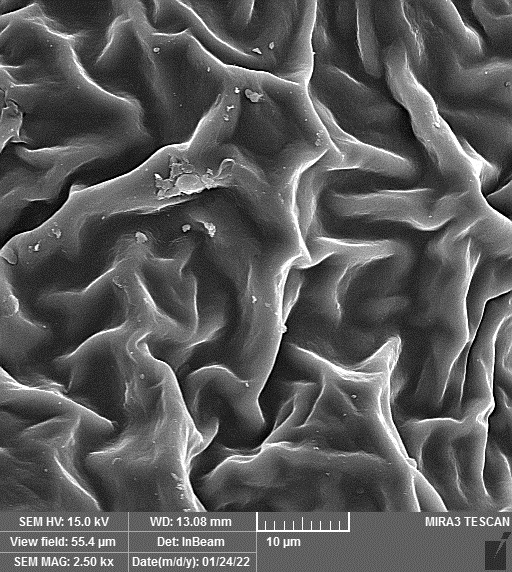	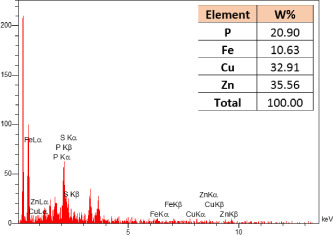
50% FC 20 mg ZnSO_4_ 20 mg ZnO-N	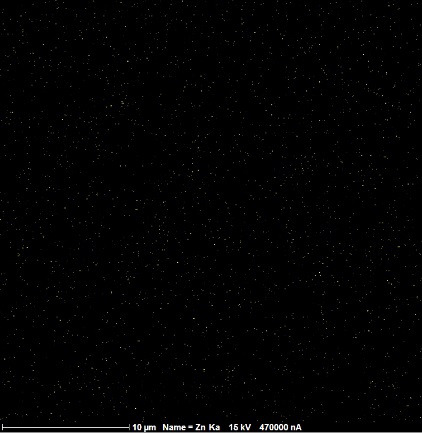	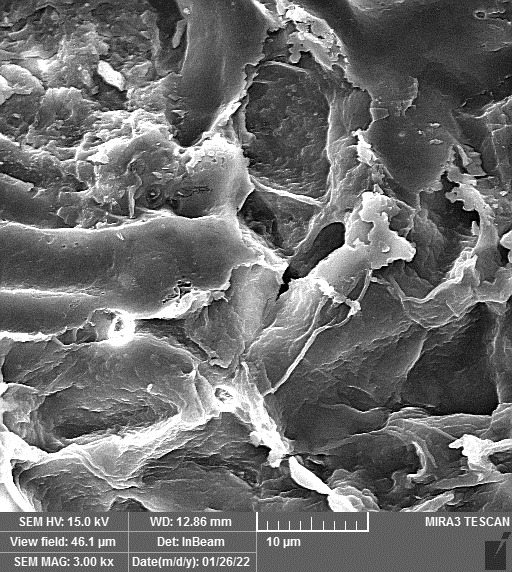	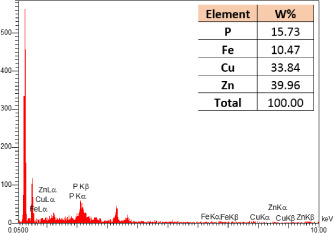
50% FC 20 mg ZnO-N	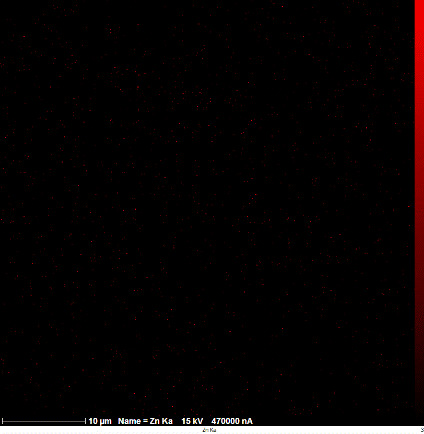	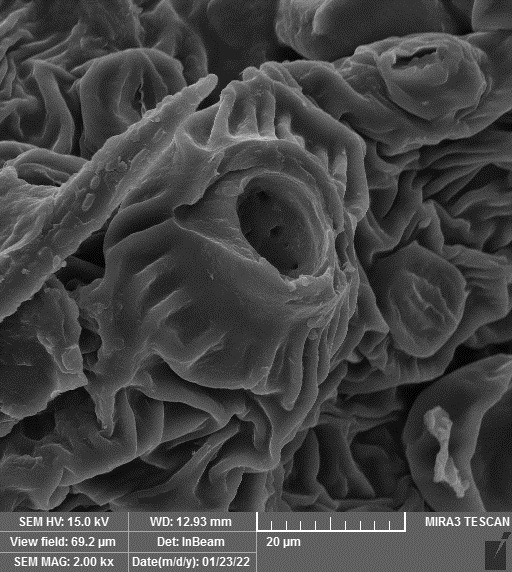	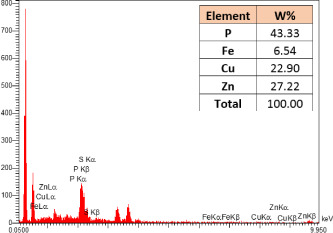

**Table 3:** Comparison of measured elements percentage with control (non-fertilized) in treated *D. kotschyi* by ZnSO4 and ZnO-N through SEM-EDX method under drought stress.

Source	P (%)	Fe (%)	Cu (%)	Zn (%)
Zn-Nanoparticle	24.85	10.47	30.30	34.37
ZnSO_4_	21.37	11.01	31.83	35.78
Control	35.86	8.27	26.35	29.52

## 4 Discussion

The present study aimed to increase the drought tolerance of *D. kotschyi* using ZnO-N and ZnSO_4_ treatments. According to the results, ZnO-N reduced EC on the plants under drought stress, while the mitigating effect of ZnSO_4_ on EC at the same conditions was less than ZnO-N. Meanwhile the highest sugar and proline content as well as the highest activity of SOD and GPO (and to some extent PPO) in *D. kotschyi* exposed to drought stress were at the same conditions observed by the application of ZnO-N. Using ZnSO_4_ increased chlorophyll and protein content and also PPO activity in this plant under drought stress.

### 4.1 Changes in physiological, biochemical contents

RWC in plants indicates the amount of water stored in the leaves and the rate of transpiration. Stress (in particular drought stress) usually reduces electrical conductivity in plants, which leads to a decline in RWC (Ahluwalia et al., 2021). In this experiment, there was a significant decrease in RWC in plants under drought stress. Nonetheless, ZnO-N and ZnSO_4_ treatments increased RWC in plants by 10% and 3%, respectively (although not statistically significant). Studies showed that using ZnO-N increases RWC in *Solanum melongena* (Semida et al., 2021) and *Triticum aestivum* (Taran et al., 2017) under drought stress. Similarly, ZnSO_4_ improved RWC in wheat (Sattar et al., 2022) and triticale (Kheirizadeh Arough et al., 2016) under stress. The Zn element affects water absorption and transport capacity in plants and decreases short-term adverse effects of environmental stresses (Kasim, 2007; Disante et al., 2010). On the other hand, regulation of gene expression affecting tolerance to environmental stress depends on the Zn element (Cakmak, 2000).

Different stresses, such as drought stress, damage cellular membrane in plants and lead to ion leakage, thereby increasing EC in the plant (Luo et al., 2012). Therefore, low EC in plants could be a criterion for resistance to environmental stresses (Blum, 2005). In this experiment, ZnO-N and to some extent ZnSO_4_ reduced EC in plants under stress, respectively compared to the control plants. According to the results, a decline in EC of plants under stress was due to the use of ZnO-N in wheat (Adrees et al., 2020) and corn (Rizwan et al., 2019).

Results indicated that drought stress led to a decrease in total chlorophyll content in *D. kotschyi*, and ZnSO_4_ was able to increase chlorophyll content in the plant under stress conditions. In support of this finding, Cakmak (2000) reported that zinc promotes chlorophyll synthesis by protecting sulfhydryl groups. In addition, it influences pigment content by creating a balance in the concentration and providing other elements involved in chlorophyll synthesis (N and Mg) (Movahhedi et al., 2017). According to various studies, Zn increases chlorophyll content under drought stress (Dimkpa et al., 2020; Faizan et al., 2021; Sun et al., 2021).

While drought treatment did not affect the sugar content of *D. kotschyi*, ZnO-N at 20 mg/l increased soluble sugar in the plants under drought stress, compared to the control plants. It is notable that Zn plays a role in carbohydrate metabolism and affects the activity of carbonic anhydrase, which regulates the CO_2_-sensing pathway and improves drought tolerance in the plant (Tewari et al., 2019). The use of Zn in plants under drought stress led to an increase in the accumulation of soluble carbohydrates and increased drought tolerance in *Phaseolus vulgaris* (Mahdieh et al., 2018) and *Sesamum indicum* (Movahhedi et al., 2017). In the corn plant, the use of ZnO-N was associated with an improvement in drought tolerance of the plant through the regulation of the activity of key enzymes in carbohydrate metabolism (Sun et al., 2021).

In this experiment, proline accumulated in the plants in response to drought stress. Proline acts as an essential osmolyte and an efficient antioxidant in regulating cell osmosis and ROS scavenging in plants under stress. Proline maintains cellular turgidity, stabilizes the structure of enzymes and proteins, and protects the plant under stress. The increase in proline content in plants can be considered an as indicator of improved drought tolerance (Ahluwalia et al., 2021). Fertilization treatment with 10 mg/l ZnO-N increased proline content in plants under drought stress. Results of some previous studies were indicative of increased proline content as a result of ZnO-N (Noohpisheh et al., 2021) and Zn (Zengin, 2006) in *Trigonella foenum-graecum* and *Phaseolus vulgaris* under environmental stresses. According to our findings, in some plants under drought stress, the increase in protein concentration was associated with higher RWC (Sandhya et al., 2010; Silva et al., 2020). Moreover, Zn led to the accumulation of proline under drought stress, and proline accumulation resulted in the protection of osmoregulation enzymes and increased drought tolerance in the plant (Hasegawa et al., 2000). The exact mechanism of the role of proline on antioxidant enzymes has not been clarified (Haque et al., 2007). However, under environmental stress such as drought, the increase in the antioxidant enzymes’ activity is usually accompanied by an increase in the proline content (Ghaffari et al., 2019).

The treatments of ZnO-N and ZnSO_4_ led to increased protein content in plants under drought stress. This could be due to the increased level of photosynthesis, RWC, chlorophyll content, and also enzymes involved in synthesizing sugar in plants (Sun et al., 2021). The decrease in EC and improvement of damages caused by ion leakage in stress conditions could also be another reason for increased protein content in Zn- treated plants (Cakmak, 2000). Sugars and proteins are two key components in regulating plant responses to stresses. On the hand, transcription, translation, protein stability, and activity are the regulatory roles of sugars in plants (Rolland et al., 2006). On the other hand, various enzymes and proteins are involved in the sugar synthesis of plants (Pfister and Zeeman, 2016). Soluble sugars as osmoprotectors, stabilize proteins and cell membranes under environmental stresses lead to different expressions of some proteins related to sugar metabolism. Moreover, sugar signals enhance plant defense responses through some enzymes (like mitogen-activated protein kinase) signaling cascade (Kocal et al., 2008). In a study, a significant positive correlation between soluble proteins and sugars in the growth of two poplar species under drought stress was reported by Yang and Miao (2010). In this respect, our findings are congruent with the reports related to the use of Zn (as a nanoparticle or sulfate) in various plants under drought stress (Patra et al., 2013; Singh et al., 2018; Dimkpa et al., 2019; Motyka et al., 2019; Bashir et al., 2021).

In our study, drought stress significantly increased the activity of SOD, GPO, and PPO antioxidant enzymes in *D. kotschyi*. Studies show that antioxidant enzyme activities increase to improve plant tolerance in stress conditions. The SOD enzyme plays an important role in the cell’s antioxidant defense system by converting peroxide radicals to hydrogen peroxide (Sharma et al., 2012). On the other hand, the PPO enzyme in the immune system of plants under environmental stress, prevents excessive reduction of electron transfer in photosynthesis (Islam et al., 2020). Meanwhile, GPO can convert H_2_O_2_ to water through oxidation (Dianat et al., 2016).

In the current research, ZnO-N increased the activities of SOD, GPO, and some extent PPO enzymes in *D. kotschyi* under drought stress. Zn is a metal component and cofactor of many enzymes and increases the activity of antioxidant enzymes under drought stress conditions (Kheirizadeh Arough et al., 2016). The use of ZnO-N plays a role in modulating the activity of various enzymes, especially those related to plant adaptation to stress (Verma et al., 2020). In some previous studies, using ZnO-N intensified SOD enzyme activities in *Salvinia natan* (Hu et al., 2014) and *Gossypium hirsutum* (Priyanka and Venkatachalam, 2016). Bashir et al. (2021) reported that SOD and PPO activities increased in *Triticum aestivum* under oxidative stress conditions by using Zn nanoparticles. In addition, iron and Zn nanoparticles increased GPO activities in *Oryza sativa* (Upadhyaya et al., 2017) and *Dracocephalum moldavica* (Moradbeygi et al., 2020) under environmental stress.

Some reports are indicating the adverse effects of nanoparticles on biological systems and cellular components. ZnO-N are also shown to have some genotoxic effects on human epidermal cells (Sharma et al., 2009). It has been found that ZnO-N can be toxic for either normal or cancer cells (Ali et al., 2012). Moreover, it can cause oxidative DNA damage in human lung cells (Ng et al., 2017). Despite some reports showing the negative impact of ZnO-N on biological systems, there are other studies indicating that the toxicity of ZnO-N is not significant to human health and the environment. It has been found that ZnO-N are non-toxic up to a certain level but can be dangerous at higher concentrations (Nagara et al., 2022). In this regard, Amuthavalli et al. (2021) showed the moderate side effect of biosynthesized ZnO-N on rats and they reported that ZnO-N could be used as a multipurpose agent in the field of biomedical research (Amuthavalli et al., 2021). ZnO-N synthesized from plants (*Melia azedarach*) showed less toxicity compared to the one synthesized through the conventional method (Dinga et al., 2022). ZnO-N have a wide range of biomedical applications (Agarwal et al., 2017) and are also used in cosmetic products such as sunscreen creams (Hong et al., 2022). Recent studies have shown that the risks and benefits of zinc oxide nanoparticles depend on various factors including the concentration of ZnO-N, the synthesis method, and the tested organism (Czyżowska and Barbasz, 2022).

Increasing the concentration of Zn in the plant could cause poisoning by disrupting the balance of other nutrients and reducing photosynthesis (Kabata-Pendias, 2010). Meanwhile, nano-fertilizers have a lower chance of creating toxicity in the plant and soil with slower and more appropriate delivery of nutrients to the plant (Solanki et al., 2015). However, nano-fertilizers could be toxic at high concentrations, for instance, ZnO-N at high concentrations could inhibit growth *in Fagopyrum esculentum* (above 1000 mg/l) (Lee et al., 2013) and Radish (above 20 mg/l) (Lin and Xing, 2007). In this experiment, low concentrations of ZnO-N (10 and 20 mg/l) were applied on *D. kotschi* with the approach of ornamental/landscape use (not as an edible plant).

### 4.2 Changes in element concentration

According to the findings, there was a significant difference in the concentration of P, Cu, Fe, and Zn elements in treated plants with ZnO-N and ZnSO_4_, compared to the control plants. However, no significant difference was observed between the treated plants with two fertilizers (ZnO-N and ZnSO_4_) in terms of the element concentration. Double interactions between P, Fe, and Zn have long been recognized in plants, such that Cu and Fe concentrations increased with an increase in Zn concentration (Fan et al., 2021). Furthermore, studies have approved the antagonistic effect of Zn and Fe with P as a nutrient in some plants (Zheng et al., 2009). Overall, P, Cu, Fe, and Zn play a significant role in drought stress tolerance in plants (Marschner, 2012; Tripathi et al., 2018; Dimkpa et al., 2019).

In a previous study (Akbari et al., 2013), fertilization with Zn reduced drought stress through decreasing H_2_O_2_ content and lipid peroxidation, which resulted from increased antioxidant enzymes (CAT, GPX, and SOD). Fe regulates the unfavorable effects of drought, salinity, and heavy elements by controlling cellular redox states and the antioxidant defense mechanism, such as catalase and superoxide dismutase (Tripathi et al., 2018). Moreover, Cu increases drought tolerance by increasing the activity of SOD, ascorbate peroxidase enzymes, and anthocyanin levels (Van Nguyen et al., 2022). In addition, P increases water uptake and maintains cellular turgidity by improving root growth, thereby regulating stomatal conductance and ultimately increasing plant photosynthesis and drought tolerance (Waraich et al., 2011).

Compared to conventional fertilizers, nano fertilizers due to their higher absorption level and slower release rate are more efficient in plant processes, including improving tolerance to stresses (Manjunath et al., 2016; Bashir et al., 2020). In general, foliar spraying by nanoparticles has many paths for absorption in plants due to the small size of the particles. Nanoparticles can enter the leaves through stomata, endocytosis, leaf hydathodes, and direct absorption (Hong et al., 2021). In this experiment, the size of the ZnO-N was not larger than 20 nm, so according to the report of Alshaal and El-Ramady (2017), compared to ZnSO_4_ it seems to be absorbed to a greater extent, and there was no limitation passing through the cell wall pores. In this regard, Rajemahadik et al. (2018) reported that nanoparticles because of dynamic properties have a high surface area, activity, and catalytic surface. Nanoparticles rapidly react and disperse and can absorb more water. These properties can make nanoparticle fertilizers perform better in plant functions than other forms of fertilizers.

The Efficiency of ZnO-N compared to ZnSO_4 (_as two Zn fertilizers) on the growth of *Zea mays* was confirmed in the study of Subbaiah et al. (2016). Also, Rossi et al. (2018) by foliar spraying of ZnO-N and ZnSO_4_ on coffee plants reported that ZnO-N significantly increased the photosynthesis rate and growth traits. Moreover, there are some studies on the positive effect of Zn-nanoparticles on enhancing tolerance to abiotic stresses (in particular drought) in *Solanum melongena* (Semida et al., 2021), *Triticum aestivum* (Taran et al., 2017), wheat (Adrees et al., 2020), corn (Rizwan et al., 2019), *Trigonella foenum-graecum* (Noohpisheh et al., 2021) and *Phaseolus vulgaris* (Zengin, 2006).

Moreover, given the equal leaf Zn level in the treated plants with ZnO-N and ZnSO_4_ at the end of the experiment (EDX analysis), the toxicity of ZnO-N was not significant than ZnSO_4_. In other words, it can be said that due to the use of low concentrations of ZnO-N (10 and 20 mg/l) in this experiment, no toxicity was observed using this fertilizer. Moreover, *D. kotschyi* can be considered as a model organism to present reduced toxicity effects of ZnO-N through supplementary experiments related to toxicity assessments (**Supplementary File 2** and **Supplementary File 3**).

## 5 Conclusion

Considering the drought problem, using compounds that could increase drought tolerance in plants plays an essential role in water management in agriculture and urban green space. This research has demonstrated that zinc nanoparticles (ZnO-N) improved drought tolerance in *Dracocephalum kotschyi* (a medicinal-ornamental endangered plant) under drought stress. Based on our findings, ZnO-N positively affected most of biochemical (sugar and proline) and physiological (electrolyte conductivity and to some extent relative water content) factors in drought conditions. Moreover, ZnO-N significantly increased the activity of antioxidant enzymes (SOD, GPO, and some extent PPO) in *D. kotschyi* exposed to drought stress. The positive effect of ZnSO_4_ application on this plant under drought stress was achieved through increased EC, RWC, chlorophyll and protein content, and also POP activity. In addition, no significant difference was observed in the concentration of P, Cu, Fe, and Zn elements in plants treated with ZnO-N and ZnSO_4_. Given the significant role of sugar and proline and also antioxidant enzymes (SOD, GPO, and to some extent PPO) in plants’ defense systems and tolerance to environmental stresses, ZnO-N seems to be more efficient compared to ZnSO_4_ in improving drought tolerance in *D. kotschyi*. Since there are some reports regarding the toxicity of ZnO-N at higher concentrations, it is recommended to use ZnO-N at a low level (less than 1000 mg/l).

## Data availability statement

The original contributions presented in the study are included in the article/**Supplementary Material**. Further inquiries can be directed to the corresponding author.

## Author contributions

All authors contributed to the study conception and design. Material preparation, experimental works and analysis were performed by ZK. The first draft of the manuscript was written by ZK. LS commented on preliminary versions of the manuscript. All authors contributed to the article and approved the submitted version.
