# Supplementary material for: ZnO nanoparticles efficiently enhance drought tolerance in Dracocephalum kotschyi through altering physiological, biochemical and elemental contents
Source: Front Plant Sci. 2023 Mar 10;14:1063618. doi: 10.3389/fpls.2023.1063618 (PMC10036906; doi:10.3389/fpls.2023.1063618)
Supplement: Supplementary file 1 [file Table_1.docx]

| **Analysis Result** | | |
| --- | --- | --- |
| Particle Size nm | 20 | - |
| Purity % | 99.8 | - |
| SSA, M2/g | 80‐120 | - |
| Loss on dry %≤ | 0.2 | - |
|  | Chemical Composition | Analysis Wt % |
| Elemental analysis | Al | 0.001 |
|  | Fe | 0.001 |
|  | Ca | 0.001 |
|  | Mg | 0.001 |
|  | Cu | N.D |
|  | Mn | 0.001 |
|  | Na | 0.001 |
|  | Co | 0.001 |
|  | Ni | 0.001 |
|  | Si | 0.001 |
|  | Pb | N.D |
|  | K | 0.001 |
|  | N | 0.004 |
|  | C | 0.001 |
|  | S | 0.003 |
|  | F.O | 0.060 |

**Supplementary Table 1** Characteristics of Zinc oxide nanoparticles, analytical technique: Inductively Coupled/Elemental Analyzer
