# Supplementary material for: ZnO nanoparticles efficiently enhance drought tolerance in Dracocephalum kotschyi through altering physiological, biochemical and elemental contents
Source: Front Plant Sci. 2023 Mar 10;14:1063618. doi: 10.3389/fpls.2023.1063618 (PMC10036906; doi:10.3389/fpls.2023.1063618)

**Mapping (X-ray-SEM) of ZN, Cu, Fe and P in in ZnO-N and ZnSO4 treatments in *Dracocephalum Kotschyi* under drought stress**




























































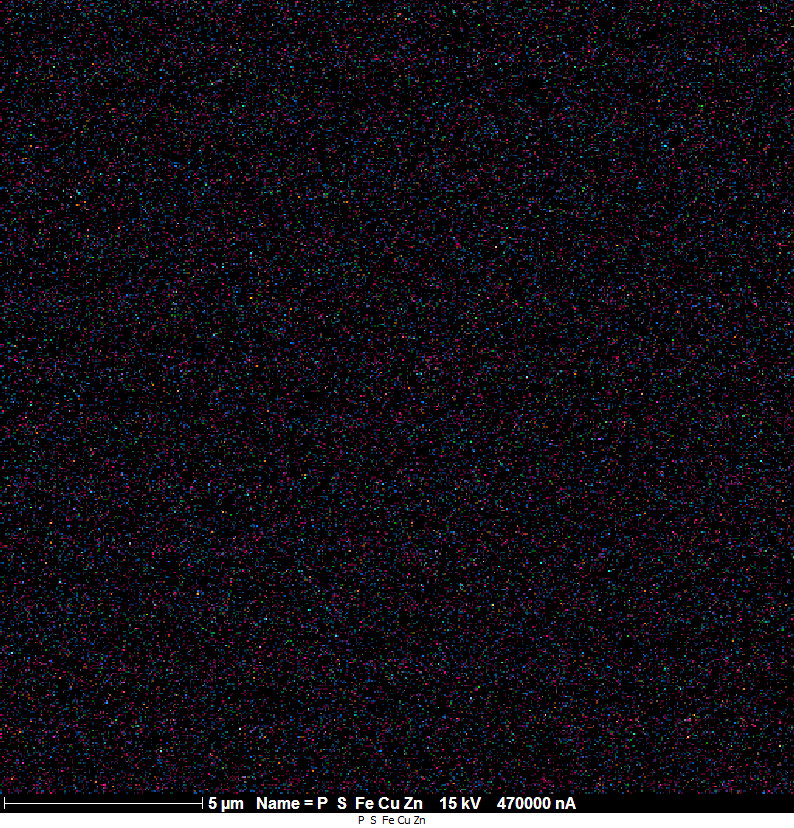

Supplement: Supplementary file 3 [file Table_3.docx]
